# Predicting oxygen requirements in patients with coronavirus disease 2019 using an artificial intelligence-clinician model based on local non-image data

**DOI:** 10.3389/fmed.2022.1042067

**Published:** 2022-11-30

**Authors:** Reiko Muto, Shigeki Fukuta, Tetsuo Watanabe, Yuichiro Shindo, Yoshihiro Kanemitsu, Shigehisa Kajikawa, Toshiyuki Yonezawa, Takahiro Inoue, Takuji Ichihashi, Yoshimune Shiratori, Shoichi Maruyama

**Affiliations:** ^1^Department of Nephrology, Nagoya University Graduate School of Medicine, Nagoya, Japan; ^2^Department of Internal Medicine, Aichi Prefectural Aichi Hospital, Okazaki, Japan; ^3^Department of Molecular Medicine and Metabolism, Research Institute of Environmental Medicine, Nagoya University, Nagoya, Japan; ^4^Artificial Intelligence Laboratory, Fujitsu Limited, Kawasaki, Japan; ^5^DX Platform Business Unit, Fujitsu Limited, Nagoya, Japan; ^6^Department of Respiratory Medicine, Nagoya University Graduate School of Medicine, Nagoya, Japan; ^7^Department of Respiratory Medicine, Allergy and Clinical Immunology, Nagoya City University Graduate School of Medical Sciences, Nagoya, Japan; ^8^Department of Respiratory Medicine and Allergology, Aichi Medical University Hospital, Nagakute, Japan; ^9^Department of Respiratory Medicine, Fujita Health University School of Medicine, Toyoake, Japan; ^10^Center for Healthcare Information Technology (C-HiT), Nagoya University, Nagoya, Japan; ^11^Medical IT Center, Nagoya University Hospital, Nagoya, Japan

**Keywords:** clinical practice, COVID-19, artificial intelligence-human collaboration, sodium chloride difference, oxygen needs

## Abstract

**Background:**

When facing unprecedented emergencies such as the coronavirus disease 2019 (COVID-19) pandemic, a predictive artificial intelligence (AI) model with real-time customized designs can be helpful for clinical decision-making support in constantly changing environments. We created models and compared the performance of AI in collaboration with a clinician and that of AI alone to predict the need for supplemental oxygen based on local, non-image data of patients with COVID-19.

**Materials and methods:**

We enrolled 30 patients with COVID-19 who were aged >60 years on admission and not treated with oxygen therapy between December 1, 2020 and January 4, 2021 in this 50-bed, single-center retrospective cohort study. The outcome was requirement for oxygen after admission.

**Results:**

The model performance to predict the need for oxygen by AI in collaboration with a clinician was better than that by AI alone. Sodium chloride difference >33.5 emerged as a novel indicator to predict the need for oxygen in patients with COVID-19. To prevent severe COVID-19 in older patients, dehydration compensation may be considered in pre-hospitalization care.

**Conclusion:**

In clinical practice, our approach enables the building of a better predictive model with prompt clinician feedback even in new scenarios. These can be applied not only to current and future pandemic situations but also to other diseases within the healthcare system.

## Introduction

In real-world clinical settings, clinicians are under time pressure for making decisions (1–3). Furthermore, during a pandemic, there is an increased urgency for clinicians to predict the disease course and make decisions in a timely fashion, even with limited data.

Nowadays, scientists and researchers use machine-learning (ML) and deep-learning (DL) models in several applications, including agriculture (4, 5), environment (6–12), text sentiment analyses (13), medicine (14), and cyber security (15–17).

Regarding clinical decision-making support systems utilizing artificial intelligence (AI), such as ML, recent studies have shown the potential of clinician involvement across the stages of design and implementation to overcome known challenges namely, increasing usability, clinical relevance, understandability, and delivering the system in a respectful manner (1, 18, 19). In the field of medical AI, recent studies have begun to explore collaborative setups between AI and clinicians. However, these studies have been mainly based on imaging data (20–28) and few studies have assessed non-image data types (29, 30). Therefore, there is scope for further research on AI-clinician collaboration involving non-image data types. Furthermore, to bridge the gap between AI and clinical implementation, it has been suggested that clinicians should train the AI model with local data, based on the needs of their patients and the hospital requirements (31).

During the ongoing coronavirus disease 2019 (COVID-19) pandemic, researchers have shown the effectiveness of ML in various fields (32–34); however, potential applications of ML for disease prevention are unclear in real-world medical settings. Furthermore, translational bioinformatics in COVID-19 research has suggested the effectiveness of ML models with customized designs (35).

Some studies have provided valuable insights on ML using predictive models built with limited data on patients with COVID-19 (36, 37), including prediction of the need for supplemental oxygen (38, 39) and big data for predicting the need for hospital admission (40). However, the performance of AI-clinician collaborative models is not yet clear. Furthermore, the efficiency of the incorporation of direct clinician perception into AI predictive models also remains unknown.

Despite limited data, clinicians can identify patients’ features and make rapid decisions from non-image data, such as vital signs, medications, and laboratory test results. However, there is a lack of appropriate tools to integrate their perception with AI predictive models for their customization. Recently, Wide Learning™ (WL), an explainable AI with ML tool, has led to the understanding of combination features from complex parameters (41). It has the potential to enable clinicians to combine their perception with AI, resulting in AI-clinician collaboration.

In this study, we created models and compared the performance of AI in collaboration with a clinician with that of AI alone in predicting the need for oxygen supplementation in patients with COVID-19, based on local non-image data.

## Materials and methods

### Research objective

The objective was to create models and compare the performance of AI in collaboration with a clinician to that of AI alone in predicting the need for supplemental oxygen in patients with COVID-19 admitted to hospital, based on local non-image data.

### Outcome

The outcome was the requirement for supplemental oxygen after admission.

### Data source

The analysis used a single set of data extracted from records of patients hospitalized in Aichi Prefectural Aichi Hospital, a 50-bed facility in Okazaki, Japan, established in October 2020 for the treatment of adult patients with mild-to-moderate COVID-19. The hospital records were collected and analyzed by physicians. All data used to develop the models were based on transparent reporting of a multivariable prediction model for individual prognosis or diagnosis (TRIPOD) (42).

### Study design

We conducted a retrospective study using hospital records. We enrolled 30 patients with COVID-19 admitted to the hospital from December 1, 2020 to January 4, 2021 who were not treated with oxygen therapy and were aged >60 years on admission. We excluded patients with COVID-19 aged <60 years and those treated with oxygen therapy on admission.

### Measurements

All patients were tested for severe acute respiratory syndrome coronavirus 2 (SARS-CoV-2) by polymerase chain reaction (PCR) on admission to the hospital, and all patients tested positive. Patients’ vital signs measured on admission were used in the analysis. Venous blood and urine samples were collected within 2 days of admission. Complete blood cell and differential leukocyte counts were performed in the clinical laboratory using an automatic analyzer XN-3000 (Sysmex, Kobe, Japan).

### Variables

Baseline demographic and clinical data were collected from patient records. The data collected included information regarding age; sex; residence before hospitalization (43) (home, hospital, or long-term care facility); comorbidities (44, 45) (number of comorbidities, cardiovascular disease, dementia, fracture, diabetes, cancer, hypertension, hyperlipidemia, chronic kidney disease, and chronic obstructive pulmonary disease); level of consciousness on admission (46); symptoms (fever, cough, and anorexia); body mass index (BMI); radiographic findings (abnormalities on chest radiograph and observation of pleural fluid on chest radiograph, checked by over two pulmonologists in our hospital); Japan Respiratory Society Community-Associated Pneumonia Severity Index score; any regular medications (number of regular medicines, dosage form with powder or liquid, and sedatives); other ongoing treatments (43, 44); intake of angiotensin-converting enzyme inhibitor/angiotensin receptor blocker, calcium channel blocker, azithromycin, β-blocker, aspirin and related drugs, non-steroidal anti-inflammatory drugs, metformin, insulin, immunosuppressants, vitamin D, hydroxychloroquine, corticosteroids, antibiotics, proton pump inhibitors, favipiravir, and remdesivir; and vital signs on admission (temperature, systolic blood pressure, diastolic blood pressure, SpO_2_, and heart rate). We collected laboratory data including hemoglobin, platelet count, blood urea nitrogen, creatinine, total serum protein, serum albumin, total cholesterol, sodium, potassium, chloride, phosphorus, calcium, uric acid, lactate dehydrogenase, creatinine kinase, total bilirubin, aspartate aminotransferase, alanine aminotransferase, glucose, serum iron, C-reactive protein, ferritin, fibrinogen, D-dimer, procalcitonin, sialylated carbohydrate antigen (kl-6), and urine sediment.

### Statistical analysis

Continuous variables were reported as the mean ± standard deviation or median and interquartile range (IQR). Patients were divided into two groups according to whether they needed supplemental oxygen after admission. To evaluate baseline characteristics and laboratory biomarkers, continuous variables were compared between the two groups using the Wilcoxon signed-rank test, and categorical variables were compared using the chi-square test. Statistical significance was set at *p* < 0.05 with a two-tailed test. All statistical analyses were performed using JMP Pro version 15.0.0 (SAS Campus Drive, Cary, NC, USA).

### Wide learning methods

WL is an ML technology developed by the Artificial Intelligence Laboratory, Fujitsu Limited, Kawasaki, Japan. This method is one of ML techniques for classification and is an extension of classic logistic regression.

WL transforms continuous variables into multiple categorical variables by dividing them into multiple value ranges using entropy in information theory for the objective variable. It examines the statistics of all possible combinations of variable categorizations up to a specified number of variables per combination. Furthermore, it selects closely related combinations from among these using a specified statistic, such as the chi-square value as the selection criterion, and creates a logistic regression model using these as explanatory variables.

Models based on logistic regression can evaluate the contributions represented by variables as regression coefficients, but WL can improve classification accuracy and explainability because it can divide the original variables into appropriate value ranges and evaluate variables that appear in combination. All combinations of variables up to length three (i.e., a maximum of three variables per combination), were evaluated using the constrained pattern mining tool to prevent overfitting (41). Although it was not a problem in this analysis because it was a small study, there is a risk of a computational explosion due to the combination, considering the practical amount of data and the number of variables per combination. The method of Iwashita et al. (41), is derived from contrast pattern mining and it uses dynamic item ordering during the pattern search to prevent computational explosion (41, 47). Therefore, WL can search for combinations of variables that would require an exponential amount of time on a worst-case basis, in less time for practical purposes.

**Model:** An ordinary linear model with statistically selected variable combinations as explanatory variables.

**Input:** Categorical and continuous data in table style (e.g., CSV format).

**Hyper parameters:** We used only one parameter as a hyper parameter: Strength of L1 regularization of logistic regression (λ), where L1 represents regularization, and λ represents the complexity parameter.

**Computational complexity:** Our method used two main processes: combination counting and logistic regression.

Combination counting was calculated as follows: O (M*^L^* × N), where O represents the computational complexity, L represents the length of the variable combination (i.e., the maximum number of variables per combination), M represents the number of variables, and N represents the number of samples.

Logistic regression depends on the regression method. We used LogitNet in the glmnet package for fitting generalized linear models via penalized maximum likelihood (48), for the logistic regression.

### AI-alone and AI-clinician models

Training procedures were used for the AI-alone and AI-clinician models. We evaluated the performance of the two models with weight, normalized mutual information (NMI), supp (ratio of positive hit samples to all positive samples), conf (ratio of positive hit samples to all hit samples.), and the chi-square value. One clinician, a nephrologist, participated in the study.

**FIGURE 1:**
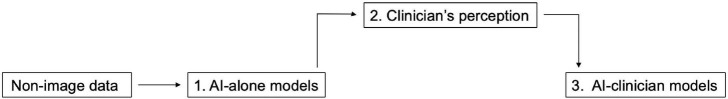
Schematic view of the development of the AI-alone and AI-clinician models. (1) AI-alone models based on non-image data. (2) Clinician perception derived from AI-alone models. (3) AI-clinician models with clinician perception.

We created AI-alone and AI-clinician models as follows (Figure 1): (1) First, we developed an AI-alone model with Wide Learning™, based on local non-image data from patients with COVID-19. (2) Second, the clinician checked the AI-alone model in a manner similar to examining patients in real clinical settings. Then, the clinician’s perception was quickly added in combination with the factors derived from the AI-alone model. For example, the AI-alone model showed Na or Cl separately, but the nephrologist combined these as the sodium chloride difference (Na – Cl), which is used for acid-base balance evaluation in real-world clinical settings. (3) Finally, we combined factors in the AI-alone model, performed retraining, and then developed AI in collaboration with the clinician, to create AI-clinician models. The input from the clinician was provided before the retraining of the AI-alone model. For example, if (Na – Cl) was added to the AI-alone model, then, retraining was performed, and an AI-clinician model was created.

### AI-non-clinician and AI-clinician-non-clinician models

For comparison, we also made AI-non-clinician models and AI-clinician-non-clinician models. The methods were the same as those used to evaluate the AI-alone and AI-clinician models, described in section “AI-alone and AI-clinician models,” above.

**AI-non-clinician model:** We used the composite variable, neutrophil-to-lymphocyte ratio (NLR) (49–51), which is a “non-clinician” variable, combined it with the AI-alone model, performed retraining, and finally created AI-non-clinician model.

**AI-clinician-non-clinician model:** We combined the variables Na – Cl, which is a “clinician” variable and the NLR, which is a “non-clinician,” variable with the AI-alone model. Then, we performed retraining and finally created the AI-clinician-non-clinician model.

### Ethics

The study was approved by the Ethics Committee of Nagoya University Graduate School of Medicine (No. 2021-0196, approval date: August 11, 2021). The requirement for obtaining informed consent was waived owing to the retrospective study design. All procedures performed were in accordance with the ethical standards of the institutional and national research committee of the institution at which the study was conducted and with the 1964 Helsinki Declaration and its later amendments or comparable ethical standards.

## Results

### Patients’ coronavirus disease 2019 characteristics and laboratory biomarkers on local non-image data

The patients’ median age was 82.6 (IQR, 74.5–88.0) years, and 76.7% of the patients were female. Baseline demographic and clinical data are presented in Table 1. Patient characteristics and laboratory biomarkers did not differ between the patients who required supplemental oxygen and the patients who did not require oxygen (Tables 1, 2).

**TABLE 1:** Clinical characteristics of patients with COVID-19 based on local non-image data.

		Received supplemental oxygen after admission		
		
	All patients (*n* = 30)	Yes (*n* = 10)	No (*n* = 20)	*p*-value
Age, median (IQR), years	82.6 (74.5–88.0)	86.0 (81.0–93.5)	81.0 (73.5–87.8)	0.137
**Sex**				0.127
Female	23 (76.7)	6 (60.0)	17 (85.0)	
Male	7 (23.3)	4 (40.0)	3 (15.0)	
**Before hospitalization**				
Home	11 (36.7)	5 (50.0)	6 (30.0)	
Hospital	10 (33.3)	2 (20.0)	8 (40.0)	
Long-term care facility	9 (30.0)	3 (30.0)	6 (30.0)	
**Coexisting disorders**				
Numbers of coexisting disorders, median (IQR)	3.7 (1.8–5.0)	4.1 (1.8–6.3)	3.5 (1.3–5.0)	0.509
Cardiovascular disease	10 (33.3)	3 (30.0)	7 (35.0)	0.784
Dementia	13 (43.3)	5 (50.0)	8 (40.0)	0.602
Fracture	8 (26.7)	3 (30.0)	5 (25.0)	0.770
Diabetes	7 (23.3)	3 (30.0)	4 (20.0)	0.542
Cancer	4 (13.3)	3 (30.0)	1 (5.0)	0.058
Hypertension	13 (43.3)	6 (60.0)	7 (35.0)	0.193
Hyperlipidemia	5 (16.7)	2 (20.0)	3 (15.0)	0.729
Chronic kidney disease	3 (10.0)	1 (10.0)	2 (10.0)	1.000
Chronic obstructive pulmonary disease	0 (0.0)	0 (0.0)	0 (0.0)	
**Condition on admission**				
Alert	19/26 (73.0)	8/9 (88.9)	11/17 (64.7)	0.186
**Symptoms**	23 (76.7)	9 (90.0)	14 (70.0)	0.222
Fever	16 (53.3)	5 (50.0)	11 (55.0)	
Cough	2 (6.7)	1 (10.0)	1 (5.0)	
Anorexia	2 (6.7)	1 (10.0)	1 (5.0)	
BMI, median (IQR)	22.1 (18.9–24.1)	24.2 (20.0–28.8)	20.5 (17.2–23.2)	0.206
**Radiologic findings**				
Abnormalities on chest radiograph	14 (46.7)	4 (40.0)	10 (50.0)	0.605
Pleural fluids on chest radiograph	0 (0.0)	0 (0.0)	0 (0.0)	
ADROP				0.881
0	3/26 (11.5)	1/9 (11.1)	2/17 (11.8)	
1	12/26 (46.2)	4/9 (44.4)	8/17 (47.1)	
2	10/26 (38.5)	4/9 (44.4)	6/17 (35.3)	
3	1/26 (3.8)	0/9 (0.0)	1/17 (5.8)	
**Regular medicines**				
Numbers of regular medicines, median (IQR)	6.1 (3.8–8.3)	5.9 (4.5–6.5)	6.3 (3.3–9.0)	0.810
Dosage forms with powder or liquid	19 (63.3)	5 (50.0)	14 (70.0)	0.284
Sedatives	5 (16.7)	1 (10.0)	4 (20.0)	0.488
ACE/ARB	9 (30.0)	4 (40.0)	5 (25.0)	0.398
Calcium channel blocker	9 (30.0)	5 (50.0)	4 (20.0)	0.091
Azithromycin	0 (0.0)	0 (0.0)	0 (0.0)	
β-blocker	0 (0.0)	0 (0.0)	0 (0.0)	
Aspirin-related	0 (0.0)	0 (0.0)	0 (0.0)	
NSAIDs	1 (3.0)	0 (0.0)	1 (5.0)	0.472
Metformin	0 (0.0)	0 (0.0)	0 (0.0)	
Insulin	0 (0.0)	0 (0.0)	0 (0.0)	
Immunosuppressants	0 (0.0)	0 (0.0)	0 (0.0)	
Vitamin D	3 (10.0)	1 (10.0)	2 (10.0)	1.000
Hydroxychloroquine	0 (0.0)	0 (0.0)	0 (0.0)	
Corticosteroids	0 (0.0)	0 (0.0)	0 (0.0)	
Anticoagulant	3 (10.0)	0 (0.0)	3 (15.0)	0.197
Statin	3 (10.0)	2 (20.0)	1 (5.0)	0.197
Antibiotics	4 (13.3)	1 (10.0)	3 (15.0)	0.704
Antidepressants	4 (13.3)	0 (0.0)	4 (20.0)	0.129
Proton pump inhibitors	9 (30.0)	2 (20.0)	7 (35.0)	0.398
Favipiravir	5 (16.7)	1 (10.0)	4 (20.0)	0.488
Remdesivir	1 (3.0)	0 (0.0)	1 (5.0)	0.472
**Vital signs on admission**				
Temperature (IQR), °C	36.4 (36.1–36.6)	36.4 (36.2–36.6)	36.4 (36.0–36.6)	0.975
Systolic blood pressure (IQR), mmHg	135 (118–146)	135 (117–148)	135 (118–146)	0.909
Diastolic blood pressure (IQR), mmHg	75 (70–80)	75 (70–80)	75 (70–81)	0.950
SpO_2_ (IQR), %	96 (95–97)	96 (95–97)	96 (96–97)	0.543
Heart rate (IQR), /min	77 (70–90)	80 (72–90)	76 (68–90)	0.413

The values shown are frequencies or proportions and percentages, unless stated otherwise.

ACE/ARB, angiotensin-converting enzyme inhibitor/angiotensin receptor blocker; ADROP, Japan Respiratory Society Community-Associated Pneumonia Severity Index; BMI, body mass index; IQR, interquartile range; NSAID, non-steroidal anti-inflammatory drug.

**TABLE 2:** Laboratory findings of patients with COVID-19 based on local non-image data.

		Received supplemental oxygen after admission			
			
Parameter	All patients (*n* = 30)	Yes (*n* = 10)	No (*n* = 20)	Reference range	*p* value
White blood cells (/μL)	4,911 (3,875–5,773)	4,868 (3,900–5,773)	4,932 (3,805–5,940)	3,300–8,400	0.890
Neutrophil count (/μL)	3,137 (2,070–3,848)	3,209 (2,185–3,853)	3,102 (1,995–3,910)	–	0.811
Neutrophils (%)	62.8 (53.1–72.0)	64.8 (57.2–72.2)	61.8 (52.3–72.4)	39.8–70.0	0.536
Lymphocyte count (/μL)	1,213 (928–1,435)	1,064 (658–1,368)	1,287 (948–1,585)	–	0.276
Lymphocytes (%)	25.7 (16.8–33.2)	22.7 (16.0–28.8)	27.3 (16.7–33.6)	25.0–48.0	0.306
Monocytes count (/μL)	490 (318–645)	562 (438–673)	454 (280–585)	–	0.180
Monocytes (%)	10.1 (6.8–12.4)	11.8 (8.3–15.0)	9.3 (6.3–11.3)	3.0–9.0	0.090
Hemoglobin (g/dL)	12.3 (11.0–13.7)	12.1 (10.5–14.0)	12.4 (11.7–13.8)	11.0–14.7	0.627
Platelets (×10^4^/μL)	20.2 (15.6–23.0)	17.8 (14.2–20.2)	21.4 (16.3–25.7)	13.0–34.0	0.118
Blood urea nitrogen (mg/dL)	19 (13–22)	20 (17–22)	19 (12–21)	8.0–22.0	0.846
Creatinine (mg/dL)	0.7 (0.5–0.9)	0.8 (0.6–1.0)	0.7 (0.5–0.8)	0.6–1.1	0.398
Total serum protein (g/dL)	6.5 (6.2–6.8)	6.5 (6.2–6.7)	6.6 (6.2–7.0)	6.7–8.3	0.739
Total cholesterol (mg/dL)	172 (75–156)	161 (133–185)	178 (157–195)	130–219	0.240
Serum albumin (g/dL)	3.3 (3.0–3.5)	3.3 (3.0–3.7)	3.3 (3.0–3.4)	4.0–5.0	0.605
Na (mmol/L)	138 (136–141)	139 (137–141)	138 (136–141)	138–146	0.513
K (mmol/L)	3.9 (3.5–4.1)	3.7 (3.5–3.9)	4.0 (3.5–4.3)	3.6–4.9	0.252
Cl (mmol/L)	103 (100–106)	102 (101–104)	103 (99–106)	99–109	0.911
Phosphorus (mg/dL)	3.4 (3.0–3.8)	3.2 (3.0–3.5)	3.5 (3.0–4.0)	3.0–4.7	0.342
Calcium (mg/dL)	8.8 (8.5–9.0)	8.8 (8.5–9.0)	8.8 (8.4–9.1)	8.4–10.2	0.978
Uric acid (mg/dL)	4.1 (3.1–5.0)	4.5 (3.3–6.3)	3.9 (2.8–4.8)	3.6–7.0	0.387
Lactate dehydrogenase (U/L)	202 (169–231)	188 (161–215)	209 (176–240)	119–229	0.234
Creatinine kinase (U/L)	107 (30–109)	81 (35–101)	119 (29–154)	62–287	0.498
Total bilirubin (mg/dL)	0.6 (0.4–0.7)	0.6 (0.4–0.8)	0.6 (0.4–0.6)	0.3–1.2	0.610
Aspartate aminotransferase (U/L)	25 (17–30)	21 (17–25)	27 (17–30)	13–33	0.224
Alanine aminotransferase (U/L)	17 (10–20)	14 (8–20)	18 (11–22)	6–30	0.296
Glucose (mg/dL)	123 (99–127)	140 (97–146)	113 (100–123)	70–109	0.182
Serum iron (μg/dL)	36 (22–51)	26 (18–29)	42 (23–55)	54–181	0.070
C-reactive protein (mg/dL)	2.3 (0.4–3.0)	2.1 (0.6–2.4)	2.4 (0.3–3.4)	0.0–0.3	0.744
Ferritin (ng/mL)	294 (160–323)	236 (86–293)	321 (211–375)	50–200	0.487
Fibrinogen (mg/dL)	422 (352–502)	384 (287–453)	441 (400–502)	200–400	0.084
D-dimer (μg/mL)	3.1 (0.6–2.6)	2.2 (0.6–2.6)	3.5 (0.9–4.0)	<1.0	0.533
Procalcitonin	0.21 (0.20–0.23)	0.18 (0.15–0.21)	0.22 (0.18–0.27)	–	0.062
KL-6 (U/mL)	304 (185–352)	228 (175–292)	340 (206–437)	105–401	0.104

KL-6, sialylated carbohydrate antigen.

### Comparison of model performance

**FIGURE 2:**
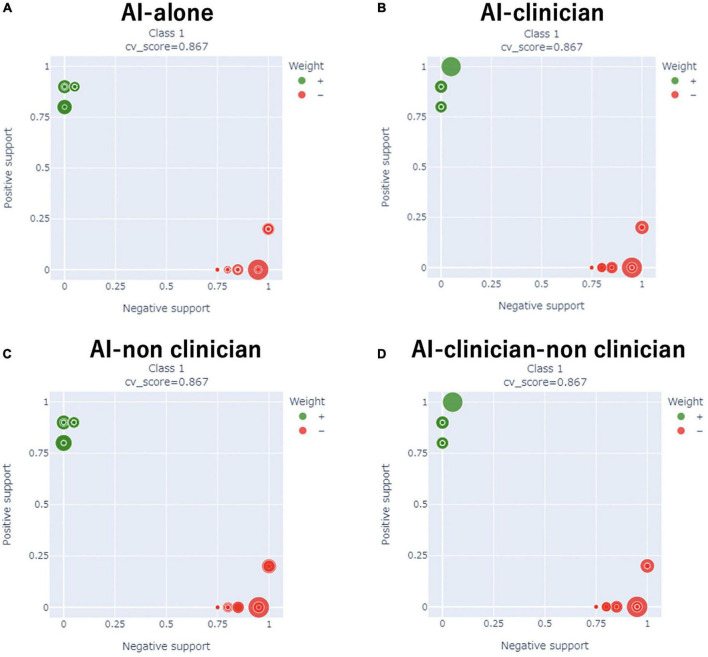
Comparisons of the performance of the AI-alone, AI-clinician, AI-non-clinician, and AI-clinician-non-clinician models to predict oxygen needs based on characteristics and laboratory biomarkers of patients with COVID-19. **(A)** AI-alone, **(B)** AI-clinician, **(C)** AI-non-clinician, and **(D)** AI-clinician-non-clinician. The green dots show the model’s performance in predicting oxygen needs in patients with COVID-19. The red dots show the model’s performance in predicting when patients with COVID-19 do not need oxygen support.

We compared the performance of the AI-alone, AI-clinician, AI-non-clinician, and the AI-clinician-non-clinician models to predict the need for supplemental oxygen based on characteristics and laboratory biomarkers of patients with COVID-19 (Figure 2). The variable combinations and model performance results are shown in Table 3. The highest weight and NMI values of the AI-clinician model were 1.4647 and 0.8245, respectively, and those of the Al-alone model were 0.9441 and 0.6490, respectively. Weight was highest in the AI-clinician model (Table 3).

**TABLE 3:** Variable combinations and model performance evaluations in AI-alone, AI-clinician, AI-non-clinician, and AI-clinician-non-clinician models for predicting the need for supplemental oxygen based on characteristics and laboratory biomarkers of patients with COVID-19.

	Models		Variable combinations	Weight	NMI	Supp	Conf	χ^2^
A	AI-alone	1	MCH ≥ 29.6 pg, lymphocyte (%) < 27.0%, eosinophils < 50.0/μL, basophils < 20/μL, APTT < 39.0 s	0.9441	0.6490	0.80	1.00000	21.8182
		2	KL-6 < 340 U/mL, TG ≥ 60 mg/dL, Cl < 106.2 mmol/L, basophil (%) < 0.4%, urine WBC negative	0.8696	0.6490	0.80	1.00000	21.8182
		3	CRP < 2.5 mg/dL, Na ≥ 136.3 mmol/L, WBC < 6,100/μL, PCT < 0.24 ng/mL, lymphocyte (%) < 39.8%	0.7472	0.7895	0.90	1.00000	25.7143
		4	CRP < 2.5 mg/dL, Cl ≥ 100.1 mmol/L, Cl < 106.2 mmol/L, WBC < 6,100/μL, neutrophil (%) ≥ 47.0%, FDP < 14.0 μg/mL	0.6927	0.7895	0.90	1.00000	25.7143
		5	TG ≥ 60 mg/dL, PCT < 0.24 ng/mL, eosinophil (%) < 0.8%, basophil < 20/μL, APTT sec < 39.0	0.4488	0.6218	0.90	0.90000	21.6750
B	AI-clinician (Na – Cl)	1	CK < 270, (Na – Cl) ≥ 33.6 mmol/L, WBC < 6,100/μL, PCT < 0.24 ng/mL, lymphocyte (%) < 39.8%	1.4647	0.8245	1.00	0.90909	25.9091
		2	CK < 270, Cl ≥ 100.1 mmol/L, Cl < 106.2 mmol/L, WBC < 6,100/μL, lymphocyte (%) < 39.8%, FDP < 14.0 μg/mL	0.6442	0.7895	0.90	1.00000	25.7143
		3	MCH ≥ 29.6, lymphocyte (%) < 27.0%, eosinophil < 50.0/μL, basophil < 20/μL, APTT < 39.0 s	0.6183	0.6490	0.80	1.00000	21.8182
		4	CRP < 2.5 mg/dL, (Na – Cl) ≥ 33.6 mmol/L, WBC < 6,100/μL, PCT < 0.24 ng/mL, lymphocyte (%) < 39.8%	0.5224	0.7895	0.90	1.00000	25.7143
		5	KL-6 < 340 U/mL, TG ≥ 60 mg/dL, (Na – Cl) ≥ 33.6 mmol/L, basophil (%) < 0.4%, urine WBC negative	0.4859	0.6490	0.80	1.00000	21.8182
C	AI-non-clinician (NLR)	1	MCH ≥ 29.6 pg, lymphocyte (%) < 27.0%, eosinophil < 50.0/μL, basophil < 20/μL, APTT < 39.0 s	1.1389	0.6490	0.80	1.00000	21.8182
		2	KL-6 < 340 U/mL, TG ≥ 60 mg/dL, Cl < 106.2 mmol/L, basophil (%) < 0.4%, urine WBC negative	0.9824	0.6490	0.80	1.00000	21.8182
		3	CRP < 2.5 mg/dL, Cl ≥ 100.1 mmol/L, Cl < 106.2 mmol/L, WBC < 6,100/μL, neutrophil (%) ≥ 47.0%, FDP < 14.0 μg/mL	0.8737	0.7895	0.90	1.00000	25.7143
		4	CRP < 2.5 mg/dL, Na ≥ 136.3 mmol/L, WBC < 6,100/μL, PCT < 0.24 ng/mL, lymphocyte (%) < 39.8%	0.7527	0.7895	0.90	1.00000	25.7143
		5	TG ≥ 60 mg/dL, PCT < 0.24 ng/mL, eosinophil (%) < 0.8%, basophil < 20/μL, APTT < 39.0 s	0.5561	0.6218	0.90	0.90000	21.6750
D	AI-clinician-non-clinician (Na – Cl, NLR)	1	CK < 270, (Na – Cl) ≥ 33.6 mmol/L, WBC < 6,100/μL, PCT < 0.24 ng/mL, lymphocyte (%) < 39.8%	1.4633	0.8245	1.00	0.90909	25.9091
		2	CK < 270, Cl ≥ 100.1 mmol/L, Cl < 106.2 mmol/L, WBC < 6,100/μL, lymphocyte (%) < 39.8%, FDP < 14.0 μg/mL	0.6422	0.7895	0.90	1.00000	25.7143
		3	MCH ≥ 29.6 pg, NLR ≥ 2.1, eosinophil < 50.0/μL, basophil < 20/μL, APTT < 39.0 s	0.6201	0.6490	0.80	1.00000	21.8182
		4	CRP < 2.5 mg/dL, (Na – Cl) ≥ 33.6 mmol/L, WBC < 6,100/μL, PCT < 0.24 ng/mL, lymphocyte (%) < 39.8%	0.5251	0.7895	0.90	1.00000	25.7143
		5	KL-6 < 340 U/mL, TG ≥ 60 mg/dL, (Na – Cl) ≥ 33.6 mmol/L, basophil (%) < 0.4%, urine WBC negative	0.4995	0.6490	0.80	1.00000	21.8182

AI, artificial intelligence; APTT, activated partial thromboplastin time; Conf, ratio of positive hit samples to all hit samples; CK, creatine kinase; CRP, C-reactive protein; FDP, fibrin degradation product; MCH, mean corpuscular hemoglobin; (Na – Cl), sodium chloride difference; NLR, neutrophil-to-lymphocyte ratio; NMI, normalized mutual information; PCT, procalcitonin; Supp, ratio of positive hit samples to all positive samples; TG, triglyceride; WBC white blood cells.

Of note, the AI-alone model included Na or Cl separately. The AI-alone model did not combine Na and Cl, i.e., the AI-alone model did not combine (Na – Cl), (Na + Cl), or (Na ÷ Cl). However, the clinician understood the variable combination, “(Na – Cl),” derived from the AI-alone model because nephrologists use (Na – Cl) for acid-base balance evaluation in clinical settings.

### Comparison between the AI-clinician model and risk factors based on published literature for predicting the need for supplemental oxygen in patients with coronavirus disease 2019

**FIGURE 3:**
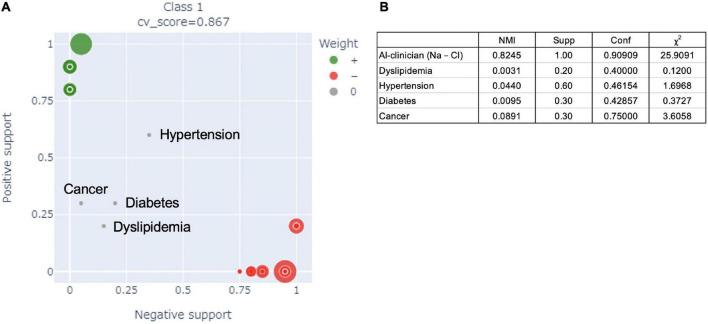
**(A)** Comparison of the performance of the AI-clinician model and risk factors in the published literature for predicting the need for supplemental oxygen based on characteristics and laboratory biomarkers of patients with COVID-19. The green dots show the AI-clinician model’s performance in predicting oxygen needs in patients with COVID-19. The red dots show the AI-clinician model’s performance in predicting when patients with COVID-19 do not need oxygen support. **(B)** Model performance evaluations in AI-clinician model and risk factors in the published literature for predicting the need for supplemental oxygen based on characteristics and laboratory biomarkers of patients with COVID-19. NMI, normalized mutual information; Supp, ratio of positive hit samples to all positive samples; Conf, ratio of positive hit samples to all hit samples; χ^2^, Chi-squared value.

Figure 3 shows a comparison of the performance of the AI-clinician model and risk factors selected from published literature for predicting the need for supplemental oxygen based on characteristics and laboratory biomarkers of patients with COVID-19. The NMI of risk factors, such as dyslipidemia, hypertension, diabetes, and cancer, selected based on published literature (44, 45) were 0.0031, 0.0440, 0.0095, and 0.0891, respectively (Figure 3B). The AI-clinician model performance evaluation values were higher than those of the risk factors selected based on published literature.

### Stepwise selection of risk factors for predicting the need for supplemental oxygen in patients with coronavirus disease 2019

Stepwise selection of risk factors to predict the need for supplemental oxygen based on characteristics and laboratory biomarkers of patients with COVID-19 are shown in Table 4. The *p*-values of Na, Cl, neutrophil count, and lymphocyte count were 0.496, 0.907, 0.803, and 0.261, respectively.

**TABLE 4:** Stepwise selection of variables predicting the need for supplemental oxygen based on clinical characteristics and laboratory biomarkers of patients with COVID-19.

Parameter	Estimate	df	Wald score/χ^2^	*p*-value
Intercept	–0.6931	1	0	>0.999
Na	0	1	0.463	0.496
Cl	0	1	0.014	0.907
Neutrophil count	0	1	0.062	0.803
Lymphocyte count	0	1	1.265	0.261

df, degrees of freedom.

## Discussion

We created a model using AI in collaboration with a clinician by rapidly combining clinician perception, such as knowledge of (Na – Cl), which is derived from variable combinations, with AI alone, on local, non-image data types, specifically patient demographic and clinical characteristics and laboratory biomarkers.

This study has two main strengths. First, it simply and rapidly added the clinician feedback to the AI-alone predictive model and improved AI-alone predictive model. Second, the clinician could consider variable combinations and the proper treatment derived from AI, such as dehydration compensation in older patients using (Na – Cl) > 33.5 mmol/L.

Real-time feedback algorithms, such as adaptive ML technology, have already been used in diverse fields, including healthcare (52), and can be used to help patients to evaluate and monitor their health risks, and alert clinicians (53–56). However, the clinician feedback and appropriate preventive treatment have not been studied. In our study, the clinician could propose treatment, such as dehydration compensation as pre-hospitalization care with AI in collaboration with a clinician predictive model, despite the limited sample size.

The discovery of (Na – Cl) > 33.5 mmol/L as a novel indicator to predict the need for supplemental oxygen in patients with COVID-19 is the main finding of this study. When clinicians, particularly nephrologists, make decisions regarding acid-base balance in practice, they use the (Na – Cl) level in the venous blood (57) and consider patients with (Na – Cl) levels of >36 mmol/L to have metabolic alkalosis. In this study, a (Na – Cl) level >33.5 mmol/L indicated that patients may have been mildly dehydrated, which may lead to asymptomatic kidney failure and hydrogen ion secretion insufficiency. This may result in a shift from severe alkalosis to slight acidosis. Arterial blood gas analysis is needed to evaluate the acid-base balance accurately; however, to prevent severe COVID-19 in older patients, dehydration compensation may be considered in pre-hospitalization care.

We speculate that the AI-clinician model is similar to patient examination by clinicians in clinical settings; therefore, clinicians would be able to easily determine the optimal treatment for patients using the AI-clinician model. Furthermore, the AI-clinician interaction might enable clinicians to find variable combinations that are different from those identified using statistical methods, leading to improved treatment in clinical settings.

However, further studies with a larger sample size, multiple clinicians, and prospective study designs, including randomized trials or prospective cohort studies, are needed. Our approach enables building of a better predictive model and ongoing application as a predictive system in real-world clinical settings. This approach could be applied not only to management of current and future infectious disease epidemics, but also to the medical management of other health-related conditions.

Our study has some limitations. First, while we evaluated the model to predict whether patients with COVID-19 would require supplemental oxygen, but we did not measure the SARS-CoV-2 viral load or the variant, which may have affected disease severity (58–61). However, we enrolled local patients with COVID-19 from December 1, 2020 to January 4, 2021, during the third wave of the COVID-19 pandemic in Japan; therefore, the SARS-CoV-2 variant is likely to have been homogeneous. Second, a recent study found that dialysis and hematologic tumors are risk factors for severe COVID-19 in older patients (62). However, no patient received dialysis or had hematologic tumors in our hospital, and further studies are warranted. This study revealed that an (Na – Cl) level of > 33.5 mmol/L is a novel indicator of disease severity in patients with COVID-19, suggesting that dehydration compensation as pre-hospitalization care may prevent severe COVID-19 in older patients with COVID-19 without dialysis or hematologic tumors. Third, while focusing on clinician perception in developing the AI model, we evaluated one clinician’s perception in this study. Further research with a larger sample size and several clinicians is needed; however, this study shows that a model using AI in collaboration with a clinician may improve the AI model performance. Questions for future research in this field include:

1. How does a clinician understand the variable combinations derived from the AI model?
2. How do various clinicians understand the variable combinations derived from the AI model?
3. How do clinicians grasp the variable combinations derived from the AI model?
4. What kind of clinician perception can develop a better AI model?
5. What kind of AI model can improve clinician perception?

In conclusion, our approach enables the development of a better predictive model by adding quick clinician perception and direct clinician feedback to the AI predictive model for decision-making. This approach could also contribute to the management of future infectious disease outbreaks and could be applied in real-world medical settings.

## Data availability statement

The raw data supporting the conclusions of this article will be made available by the authors, without undue reservation.

## Ethics statement

This study was approved by the Ethics Committee of Nagoya University Graduate School of Medicine (No. 2021-0196, approval date: August 11, 2021). Written informed consent for participation was not required for this study in accordance with the national legislation and the institutional requirements.

## Author contributions

RM: conception and planning, data acquisition, data analysis, critical input on the design of the study and revision of the manuscript, manuscript drafting, interpretation of data, and patient treatment. SF and TW: conception and planning, data analysis, critical input on the design of the study, and revision of the manuscript. YuS, YK, SK, TY, TIn, and TIc: data acquisition, critical input on the design of the study and revision of the manuscript, and patient treatment. YoS: critical input on the design of the study and revision of the manuscript, manuscript drafting, and advice on concept. SM: critical input on the design of the study and revision of the manuscript, manuscript drafting, and interpretation of data. All authors involved in drafting, reviewing, and approving the final manuscript.

## Abbreviations

ACE/ARB: angiotensin-converting enzyme inhibitor/angiotensin receptor blocker
ADROP: Japan Respiratory Society Community-Associated Pneumonia Severity Index
AI: artificial intelligence
BMI: body mass index
COVID-19: coronavirus disease 2019
IQR: interquartile range
KL-6: sialylated carbohydrate antigen
(Na – Cl): sodium chloride difference
NMI: normalized mutual information
NSAID: non-steroidal anti-inflammatory drug
SARS-CoV-2: severe acute respiratory syndrome coronavirus 2.
